# The *Glaesserella parasuis* phosphoglucomutase is partially required for lipooligosaccharide synthesis

**DOI:** 10.1186/s13567-020-00822-9

**Published:** 2020-07-31

**Authors:** Saixiang Feng, Aihua Chen, Xiaobing Wang, Zhichao Pan, Siqi Xu, Huiwen Yu, Bin Zhang, Ming Liao

**Affiliations:** 1 College of Veterinary Medicine, South China Agricultural University, Guangzhou, China; 2 College of Life Science and Technology, Southwest Minzu University, Chengdu, China

**Keywords:** *Glaesserella parasuis*, lipooligosaccharide, phosphoglucomutase, biofilm

## Abstract

Lipooligosaccharides (LOSs) are virulence determinants of *Glaesserella parasuis*, a pathogen of the respiratory tract of pigs. We previously reported that disruption of the *galU* or *galE* gene in *G. parasuis* results in increased sensitivity to porcine serum, indicating that the galactose catabolism pathway is required for polysaccharide formation in *G. parasuis*. Here, we evaluated the role of the *HAPS_0849* gene in LOS synthesis. The *G. parasuis* SC096 *HAPS_0849* mutant produced a highly truncated LOS molecule, although a small fraction of intact LOS was still observed, and this mutant was found to be more sensitive to serum than the parental strain. HAPS_0849 was overexpressed and purified for biochemical assays, and this protein exhibited phosphoglucomutase (PGM) activity. Heterologous expression of a *pgm* gene from *Escherichia coli* in the *HAPS_0849* mutant led to restoration of the wild-type LOS glycoform, further demonstrating the PGM function of HAPS_0849 in *G. parasuis*. The autoagglutination and biofilm formation ability of this strain were also investigated. Disruption of *HAPS_0849* led to an increased tendency to autoagglutinate and form more biofilms, and these enhanced phenotypes were observed in the absence of glucose. In addition, LOSs from *HAPS_0849*, *galU* and *lgtB* mutants had similar truncated glycoforms, while LOSs from the *galE* and *lex*-*1* mutants exhibited another type of defective LOS pattern. These findings imply that HAPS_0849 may function upstream of GalU in the generation of glucose 1-phosphate. In conclusion, our results preliminarily described the functions of *HAPS_0849* in *G. parasuis*, and this gene was partially required for LOS synthesis.

## Introduction

*Glaesserella parasuis* is a pathogen of the upper respiratory tract of conventional swine herds and the aetiological factor of Glässer’s disease, causing fibrinous polyserositis, arthritis, and meningitis. *G. parasuis* has emerged as one of the major causes of high mortality rates in piglets, resulting in significant economic losses to the swine industry worldwide [1, 2]. The complement system is part of the innate immune system and is a first line of defence. The cascade of reactions that follows complement activation can lead to the formation of the membrane attack complex (MAC), resulting in lysis of the pathogen [3]. The complement susceptibility of *G. parasuis* strains of different clinical origins is highly variable. In general, isolates from the nares of healthy piglets are sensitive to the bactericidal effect of the complement system, while strains from systemic diseases of swine tend to be resistant to serum [4]. In a previous study, the *capD* gene, encoding a capsular polysaccharide biosynthesis protein, was identified as a virulence-associated factor, indicating that surface polysaccharides may participate in virulence, particularly in the context of serum resistance in *G. parasuis* [5].

Bacterial polysaccharides (PCs), including capsular polysaccharides (CPSs) and lipooligosaccharides (LOSs), are widespread structures found on the cell surfaces of a broad range of *Haemophilus* species and have been confirmed as major complement resistance factors in these bacteria [6–10]. Capsules were previously observed in some *G. parasuis* strains by precipitation with hexadecyl trimethylammonium bromide (CTAB), and the presence of acidic polysaccharides in some types of capsules was observed. However, capsules from other strains did not coprecipitate with CTAB, suggesting that the compositions of these capsules may be quite different or that CPS is not synthesized by these strains [11]. This finding may be attributed to the genetic diversity of the capsular biosynthetic locus in strains of different serotypes [12]. Furthermore, three heptosyltransferase genes (*opsX*, *rfaF*, and *waaQ*) are required for lipooligosaccharide (LOS) synthesis. Disruption of these genes resulted in serum-sensitive mutants, indicating that LOSs are also needed for virulence of *G. parasuis* [13].

**Figure 1:**
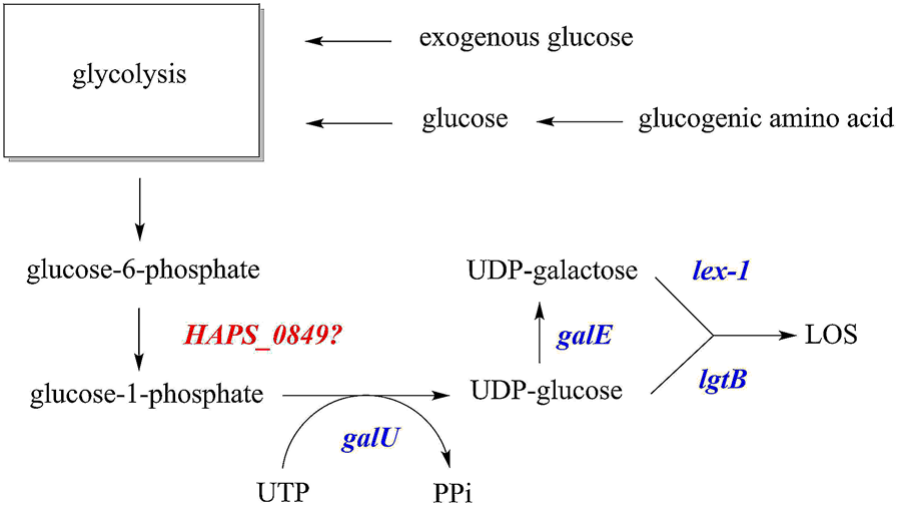
**Schematic representation of UDP-sugars biosynthesis in*G. parasuis***. The genes involved in polysaccharide synthesis with experimental evidence are marked in blue. The *HAPS_0849* gene, annotated as a phosphoglucomutase, is marked as red.

The biosynthesis of surface polysaccharides is closely related to carbohydrate metabolism in pathogens [14–16]. In our previous study, two galactose metabolism-relevant genes, denoted *galU* and *galE*, were found to affect virulence and biofilm formation in *G. parasuis* SC096 [17]. The metabolism of galactose may affect the synthesis of CPS or LOS, as *galU* and *galE* mutants are notably more sensitive to killing by porcine serum than their parent strain. As shown in Figure 1, exogenous glucose or amino acids can be converted to glucose 1-phosphate (G1P) by glycolysis then converted to uridine diphosphate glucose (UDP-glucose) by UDP-glucose pyrophosphorylase (GalU) and further converted to UDP-galactose by UDP-glucose-4-epimerase (GalE). These monosaccharide derivatives can then be utilized for the synthesis of various polysaccharides by glycosyltransferases, such as *lgtB* and *lex*-*1,* as described in our previous study [18]. To date, the genome sequences of many *G. parasuis* strains have been determined [19–22], and several virulence factors involved in the evasion of immune responses have been identified [5, 13, 17, 23–25]. However, the correlation between UDP-sugars biosynthesis and virulence has yet to be further investigated. The gene *HAPS_0849* (NCBI accession no. ACL32480.1) in the sequenced strain SH0165 is annotated as encoding phospho-sugar mutase. This mutase may be an upper-pathway enzyme involved in the generation of G1P, which is further used for LOS synthesis by GalU as described above (Figure 1). The phosphohexomutase family includes several homologous enzymes that catalyse the reversible conversion of 1-phospho-sugar substrates to 6-phospho-sugar substrates. The proteins in this superfamily play important roles in carbon source metabolism in bacteria. At least three related proteins constitute the majority of this superfamily: the highly specific phosphoglucomutase (PGM) protein, which uses only glucose as a substrate, the mannose specific phosphomannomutase (PMM) protein, and the less specific PMM/PGM protein, a bifunctional protein that can use either mannose or glucose [26]. *HAPS_0849* is homologous to the gene *pgmB*, which is predicted to encode a PGM and is involved in LOS synthesis in *Haemophilus influenzae* [27]. However, the biochemical functions of these genes in *Haemophilus* species are still unclear. Importantly, the correlation between PGM and the source of G1P mentioned above, as well as the roles of PGM in *G. parasuis*, remain poorly understood. Surface polysaccharides of *G. parasuis* are important virulence factors and key antigens for vaccine development. The pathway *pgm*-*galU*-*galE* may be a key node for these polysaccharides biosynthesis based on our studies although it is yet to be investigated further. How these polysaccharides synthesis affected by metabolism, and how to improve the yield of these key antigens? Understanding of *G. parasuis pgm* may provide some hints to answer these questions.

In the present study, we identified the gene *HAPS_0849*, encoding a PGM, as a virulence factor in *G. parasuis*. Phenotypes of its mutant affected by concentration of glucose are different from that of other reported LOS mutants in *G. parasuis*, and interestingly the *HAPS_0849* gene is partially required for lipooligosaccharide synthesis. To our best knowledge, it may be the first report to show these findings in *Pasteurellaceae* species and even in other bacteria. We have attempted to explain the relevance between PGM and polysaccharide synthesis in *G. parasuis* and pinpoint the molecular basis of this link.

## Materials and methods

### Bacterial strains, plasmids, and growth conditions

The bacterial strains and plasmids used in this study are listed in Additional files 1 and 2. *Escherichia coli* strains were routinely grown in Luria–Bertani (LB) broth or on LB agar plates at 37 °C. *G. parasuis* strains were cultured on trypticase soy agar (TSA) or trypticase soy broth (TSB) with or without dextrose (BD Biosciences, USA) and supplemented with 0.005% nicotinamide adenine dinucleotide (NAD) (Sigma Aldrich, USA) and 5% inactivated bovine serum at 37 °C in 5% CO_2_ as previously described [17]. When required, the media were supplemented with kanamycin (30 µg/mL) or gentamicin (30 µg/mL).

### Molecular biology methods

Plasmid isolation was performed using the QIAprep Spin Miniprep Kit (Qiagen, Germany). Restriction enzymes were purchased from New England BioLabs Inc. T4 DNA ligase, Taq polymerase and high-fidelity DNA polymerase were purchased from Takara Bio Inc., Japan. Restriction enzyme digestion, ligation, and transformation of *E. coli* were performed using standard methods. Genomic DNA was extracted with the E.Z.N.A. Bacterial Genomic DNA Kit (Omega Bio-Tek, USA). PCRs were used to produce DNA fragments and evaluate mutants.

### Construction of plasmids and strains

The primers used in this study are listed in Additional file 3. Mutants were constructed as previously described [17] with some modification. To mutate the *G. parasuis HAPS_0849* gene, regions of homology flanking the *HAPS_0849* locus were amplified by PCR using SC096 genomic DNA as a template. The upstream region was amplified using primers P1 and P2, while the downstream fragment was amplified using primers P3 and P4. The KanR gene was amplified from the plasmid template pK18mobsacB with primers P11 and P12, and these three fragments were joined by overlap PCR with the primer pair P1 and P4. This PCR product was then cloned into pMD19-T (Takara Bio Inc., Japan) to generate pSF251. The constructed plasmid was then used for natural transformation, and the *HAPS_0849* mutant was generated with the method described previously [23]. Briefly, bacteria were cultivated overnight at 37 °C on TSA plates and then washed and resuspended in TSB supplemented with newborn calf serum and NAD at 5 × 10^9^ CFU/mL. A 20-µL bacterial suspension was pipetted onto TSA supplemented with inactivated serum and coenzyme and spread onto a small area. One microgram of the plasmid DNA was then added and mixed, followed by cultivation for 5 h at 37 °C. Cultures were scraped off and plated on TSA with 30 µg/mL kanamycin and then incubated at 37 °C for 2 days. Positive clones were evaluated by PCR and sequencing. The *wclP* and *wcaJ* mutant strains were constructed by a similar method with the primers P5-P8 and P33-P36 listed in Additional file 3.

Complemented strains were constructed using the method published previously [28] with some modifications. Intact *HAPS_0849* was amplified with primers P13 and P14. This PCR fragment was digested with SalI and SphI, subjected to agarose gel electrophoresis followed by purification with the QIAquick Gel Extraction Kit (Qiagen, Germany), and ligated into pSF116, a complementation vector for inserting target genes between *ompP5* and *msrB*, to generate pSF254. This recombinant plasmid was transformed into the *HAPS_0849* mutant by natural transformation, and selection of the complemented *G. parasuis* Δ*HAPS_0849* strain (SF415) was accomplished using TSA containing gentamycin. Transformants were confirmed by PCR and sequencing using primers P5 and P10. The Δ*wclP* and Δ*wcaJ* complemented strains (SF416 and SF417, respectively) were constructed using a similar method as that described above with the corresponding primers (Additional file 3).

The Δ*HAPS_0849* strain expressing *E. coli pgm* was constructed as follows: the *pgm* gene fragment was amplified from *E. coli* MG1655 genomic DNA by PCR with primers P19 and P20. After purification and digestion with SalI and SphI, *Ecpgm* was ligated into pSF116 to create pSF257. This plasmid was then transformed into the Δ*HAPS_0849* strain by natural transformation and selected using gentamicin (Gm). Positive clones were confirmed by PCR amplification and sequencing using primers P5 and P10.

To construct the *G. parasuis* strain overexpressing *HAPS_0849*, complete open reading frame of *HAPS_0849* was amplified by PCR using the primer pair P39 and P40. After gel extraction, the PCR product and pET28(a) vector were digested with NdeI and HindIII, gel purified, and ligated together to corresponding sites using DNA ligase, generating the expression plasmid pSF258. The correct recombination was confirmed by PCR and sequencing.

### Overexpression and purification of *G. parasuis HAPS_0849*

pET28-*HAPS_0849* was transformed into *E. coli* BL21 (DE3) for overexpression. A single colony was selected and cultured in a flask overnight and then used to inoculate 200 mL of LB broth supplemented with kanamycin at a concentration of 50 µg/mL. The cells were cultivated at 37 °C, and then isopropyl-β-d-thiogalactoside (IPTG) was added at a concentration of 1 mM when the absorbance OD_600_ reached 0.8. The induced cells were incubated for another 4 h and then centrifuged to obtain a bacterial pellet. Next, the cells were resuspended in 1/5 volume of lysis buffer (50 mM NaH_2_PO_4_, 300 mM NaCl, 10 mM imidazole, pH 8.0) and lysed by ultrasonic treatment for 20 min. Supernatants were collected after centrifugation at 13,400 × *g* for 10 min, and then the lysate was filtered with a 0.22 µm Millipore polyethersulfone filter. Nickel-nitrilotriacetic acid (Ni-NTA) matrices (GE Healthcare) were used for protein purification. The Ni-NTA column was preequilibrated with ten volumes of lysis buffer, and then the samples were loaded onto the column by gravity. After washing with ten volumes of washing buffer (50 mM NaH_2_PO_4_, 300 mM NaCl, 20 mM imidazole, pH 8.0), target proteins were then eluted with 2 mL of elution buffer (50 mM NaH_2_PO_4_, 300 mM NaCl, 500 mM imidazole, pH 8.0) in 4 fractions. The purified proteins were analysed by sodium dodecyl sulfate-polyacrylamide gel electrophoresis (SDS-PAGE). The fractions with pure protein were pooled and dialyzed against dialysis buffer (lysis buffer without imidazole). The protein concentrations were determined with a Quick Start Bradford Protein Assay kit (Biorad, USA) and then diluted to 1 mg/mL with dialysis buffer for further enzyme activity analysis.

### Phosphoglucomutase activity assay

The activity of HAPS_0849 was confirmed with a Glucose-6-Phosphate Assay Kit (Sigma, MAK014) according to the manufacturer’s manual, with some modifications. Briefly, 10 µL of 1 mM G1P was added to a 96-well plate, and then G6P assay buffer was added to each well to bring the volume to 50 µL. Another 50-µL reaction mix containing 45 µL of G6P Assay Buffer, 2 µL of G6P Enzyme Mix, 2 µL of G6P Substrate Mix and 1 µL of protein sample or phosphate buffered saline (PBS) as a negative control was prepared. Next, 50 µL of the reaction mix was added to 50 µL of the 1 mM G1P solution described above and mixed well by pipetting. The reaction was incubated for 30 min at room temperature, and then the absorbance was measured at 450 nm (A450). To eliminate the effect of background NADH or NADPH, a blank sample was prepared for each sample by omitting the glucose-6-phosphate enzyme mix. The blank readings were subtracted from the sample readings. Activity was calculated as U (units) = (A_s_ − A_b_)/(T_1_ − T_0_) × df (where A_s_, A450 of sample after 30 min; A_b_, A450 of sample without G6P enzyme mix as a blank; T_1_, time reaction was stopped; T_0_, time reaction began; df, dilution factor of protein). Phosphoglucomutase from rabbit muscle (Sigma Aldrich, USA) was used as positive control.

### Autoagglutination assay

The ability of *G. parasuis* strains to autoagglutinate was tested by a modified method as previously reported [17]. Briefly, *G. parasuis* strains were cultured overnight in 100 mL of TSB broth with or without glucose and supplemented with 0.005% NAD and 5% inactivated serum and diluted to an optical density at 600 nm of one. The bacterial cultures were then transferred to 15 mL tubes and allowed to remain static at room temperature. The turbidity of the culture was determined every 60 min for 12 h.

### Biofilm formation assay

The biofilm formation test was performed as previously reported with some modifications [29, 30]. Briefly, 20 µL of overnight culture of the wild-type strain and its mutants were inoculated into 2 mL of fresh TSB broth containing NAD and inactivated serum with or without glucose in 6-well plates, and then incubated for 24 h or 72 h at 37 °C. Supernatants were removed by pipetting; then, biofilms were rinsed with sterile distilled water and stained with 0.1% crystal violet for 30 min. Afterwards, excess staining was gently washed off with dH_2_O, and the biofilms were dried. Bound crystal violet was dissolved in 1 mL of 33% (v/v) acetic acid, and 200 µL of solution was transferred to a 96-well microtiter plate. Then, the optical density was measured at 600 nm. All experiments were performed independently three times.

### Serum sensitivity assay

The serum bactericidal assay was carried out as previously described with 50% porcine serum, which was also obtained from a previous study [17]. Serum inactivated at 56 °C for 30 min was used as a control in all experiments. The wild-type strain and mutants were cultured to mid-logarithmic phase, and then, 100 μL of porcine serum or inactivated serum was added to 100 μL of a bacterial dilution (10^8^ CFU), followed by incubation at 37 °C for 1 h. The bacterial cells were then serially diluted tenfold and plated on TSA plates supplemented with serum and NAD. The plates were cultivated at 37 °C in an atmosphere containing 5% CO_2_ for 48 h. The survival percentage was determined according to the ratio of bacterial colonies in natural serum to those in heat-inactivated serum. All strains were tested in at least three independent experiments.

### Preparation of LOSs and electrophoresis

Bacterial polysaccharides were prepared from *G. parasuis* strains as described previously [18]. Bacterial cells were grown on TSA supplemented with NAD and 5% newborn calf serum (NBS) and washed with PBS, and LOSs were extracted with a concentration of 50% phenol at 70 °C for 15 min. The resulting aqueous phase was dialyzed against distilled water until phenol was removed (molecular weight cutoff, 1000 Da). The samples were separated by 15% SDS-PAGE, and the gels were visualized using silver staining.

### Statistical analysis

All experiments were performed at least three times, and the standard deviations were calculated with GraphPad Prism. Student’s t-test was used to evaluate the significance of differences. A *P*-value < 0.05 was considered statistically significant.

## Results

### The *HAPS_0849* mutant has defects in LOS synthesis and is sensitive to serum

**Figure 2:**
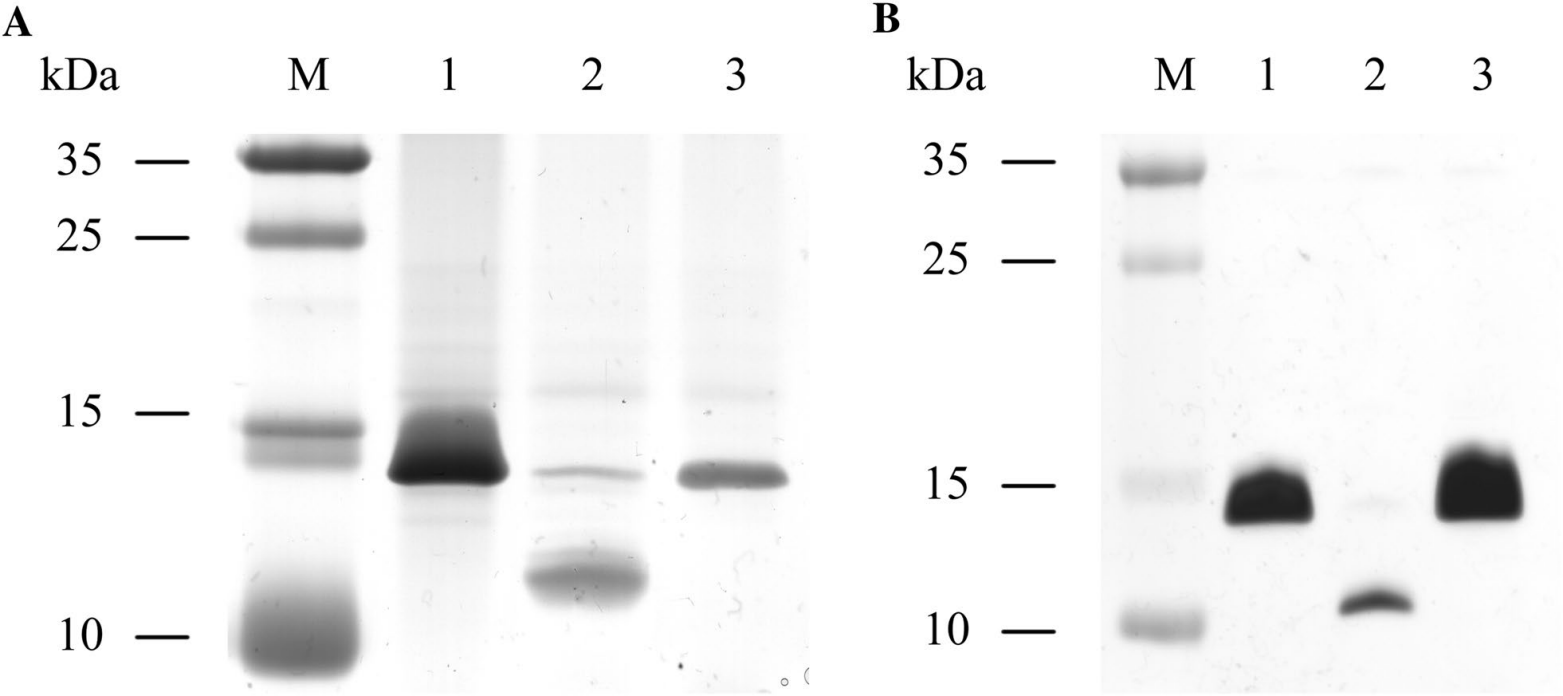
**SDS-PAGE analysis of LOSs extracted from*G. parasuis*SC096, Δ*HAPS_0849*and the*HAPS_0849*complementation strain. A** LOSs were from strains cultured in TSB with 0.25% glucose. Lane M, protein molecular marker; lane 1, wild type; lane 2, Δ*HAPS_0849* mutant; lane 3, complemented strain, Δ*HAPS_0849*-comp. **B** LOSs were from strains cultured in TSB without glucose. Lane M, protein molecular marker; lane 1, wild type; lane 2, Δ*HAPS_0849* mutant; lane 3, complemented strain, Δ*HAPS_0849*-comp.

To investigate the function of *HAPS_0849*, a kanamycin resistance cassette with flanked regions of this gene was used to disrupt *HAPS_0849* on the chromosome of strain SC096, and the generation of the complemented strain is described in “Materials and methods” section (Additional file 4). Our previous studies have shown that the heptosyltransferase genes *opsX*, *rfaF*, and *waaQ* are required for the transfer of heptoses I, II, and III, respectively, in *G. parasuis* [13], and these heptoses are derived from UDP-glucose, which is generated through the *galUE* pathway. Therefore, we sought to determine whether *HAPS_0849* was involved in lipooligosaccharide synthesis and participated in the generation of UDP-glucose (Figure 1). The LOSs from the *HAPS_0849* mutant, along with the wild-type strain SC096, were visualized by SDS-PAGE and silver staining. Figure 2A shows that SC096 expressed a single LOS band (lane 1), and Δ*HAPS_0849* produced two types of LOS glycoforms (lane 2) when these strains were cultured in TSB with 0.25% glucose. The principal LOS glycoform of Δ*HAPS_0849* migrated faster than the wild-type LOS, which suggests that the *HAPS_0849* mutant synthesized a highly truncated LOS molecule. Notably, Δ*HAPS_0849* also partially produced intact LOS, with a banding pattern corresponding to that of the wild-type LOS. However, the proportion of complete LOS was obviously lower when this mutant was cultured in TSB without glucose (Figure 2B, lane 2). In addition, LOS glycoforms from the complemented *HAPS_0849* mutant displayed a migration profile that was consistent with the migration profile of LOS from the wild-type strain (lane 3 in Figures 2A, B).

**Figure 3:**
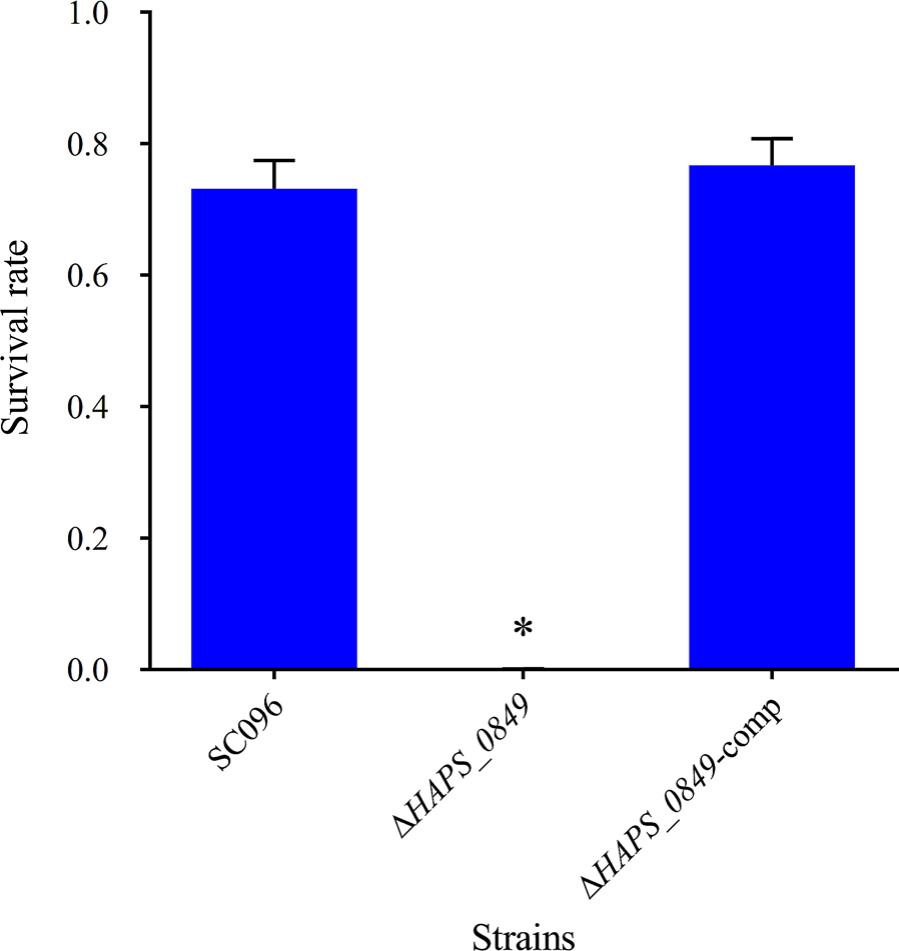
**Survival of the*HAPS_0849*mutant treated with 50% porcine serum.** The Δ*HAPS_0849* mutant strain showed significantly increased sensitivity to 50% porcine serum compared to the wild-type strain SC096 (*P < 0.01). The serum resistance phenotype was restored in the complemented strain. Error bars are standard errors of the means (SEM) from three independent experiments.

LOSs and outer membrane proteins have been proposed to play a role in serum resistance in *G. parasuis* [13, 23]. To investigate whether *HAPS_0849* also contributes to serum resistance, the sensitivity of Δ*HAPS_0849* to 50% swine serum was investigated. The survival rates of wild-type strain SC096, *HAPS_0849* mutant and complemented strain were 73.16 ± 4.26%, 0.052 ± 0.046% and 76.68 ± 4.06% respectively. The bactericidal activity of swine serum with the *HAPS_0849* mutant was significantly greater than that of the parent strain *G. parasuis* SC096 (*P* < 0.01), whereas the serum resistance phenotype was restored in the complemented strain (Figure 3). It should be noted that there was negligible variance in the doubling times between Δ*HAPS_0849* and the wild-type strain SC096 (Additional file 5). These results suggest that *HAPS_0849* was partially required for LOS synthesis and that the *HAPS_0849* mutant was sensitive to the bactericidal effects of porcine serum.

### *HAPS_0849* encodes a phosphoglucomutase

**Figure 4:**
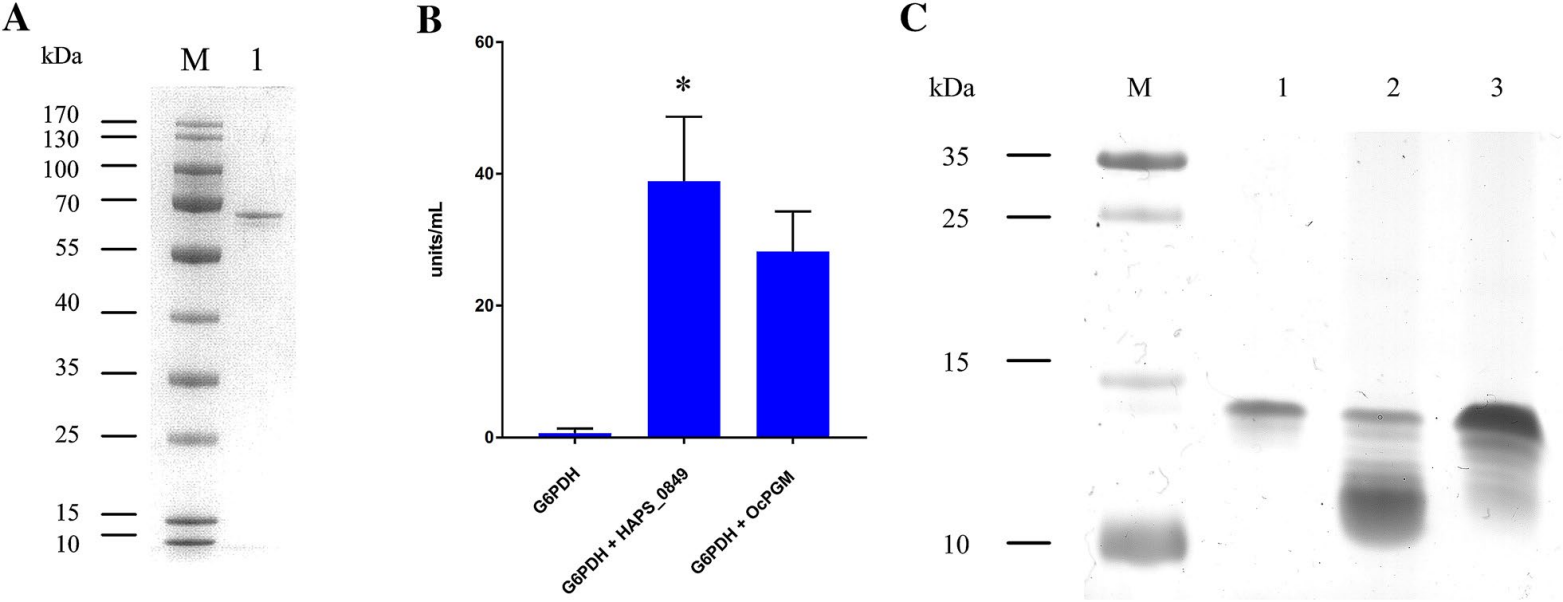
**Analysis of*HAPS_0849*by activity assay and gene complementation with*E. coli pgm*. A** Expression and purification of HAPS_0849 in *E. coli* BL21 (DE3). Lane M, protein molecular marker; lane 1, purified and diluted HAPS_0849. **B** PGM activity assay described in “Materials and methods” section. G1P was used as the substrate; the reaction started only with 6-phosphoglucose dehydrogenase (G6PDH) was set as the negative control (NC), OcPGM was from rabbit muscle as positive control. In comparison with the NC, HAPS_0849 exhibited significant PGM activities in the enzyme-linked reaction (*P < 0.05). Bars represent the mean ± standard error of three independent experiments. **C** LOS profiles of the *G. parasuis* SC096, Δ*HAPS_0849* and *Ecpgm*-expressing strains. Lane M, protein molecular marker; lane 1, wild-type; lane 2, Δ*HAPS_0849* mutant; lane 3, Δ*HAPS_0849*-*ompP5*::*Ecpgm*.

In order to confirm the biochemical functions of HAPS_0849, we constructed a pET28-*HAPS_0849* plasmid and transformed it into *E. coli* strain BL21 (DE3) for overexpression. After IPTG induction and ultrasonication, the concentration of target protein in the soluble fraction was high. Afterwards, we purified the proteins from the supernatant solution directly with Ni-NTA resin. After affinity chromatography, the HAPS_0849 protein was diluted to the appropriate concentration, analysed by SDS-PAGE (Figure 4A) and further confirmed by matrix-assisted laser desorption ionization time of flight mass spectrometry (MALDI-TOF MS) analysis (data not shown). For enzyme activity analysis, a modified phosphoglucomutase assay was performed as described in “Materials and methods” section. The purified protein had a significant ability (*P* < 0.05) to convert glucose 1-phosphate to glucose 6-phosphate generation compared to the negative control (Figure 4B), indicating that HAPS_0849 may have phosphoglucomutase activity.

The *E. coli pgm* gene (*Ecpgm*) encodes phosphoglucomutase, which is specifically essential for the conversion of glucose 6-phosphate to glucose 1-phosphate [31]. To further assess whether *G. parasuis* HAPS_0849 is a phosphoglucomutase, we constructed an *Ecpgm*-expressing strain derived from the *HAPS_0849* mutant (Additional file 6). LOSs were extracted and analysed by electrophoresis; as shown in Figure 4C, a principal LOS glycoform from the *pgm*-expressing strain was disrupted in *HAPS_0849* (lane 3), with an apparent molecular weight equivalent to that of the major LOS profile expressed by the wild-type strain (lane 1). Moreover, a small LOS glycoform was mainly observed in Δ*HAPS_0849*, as predicted (lane 2). Complementation of the *HAPS_0849* mutant with the *Ecpgm* gene also implied that HAPS_0849 may mainly function as a phosphoglucomutase in *G. parasuis*.

### Disruption of *HAPS_0849* enhances autoagglutination and biofilm formation

**Figure 5:**
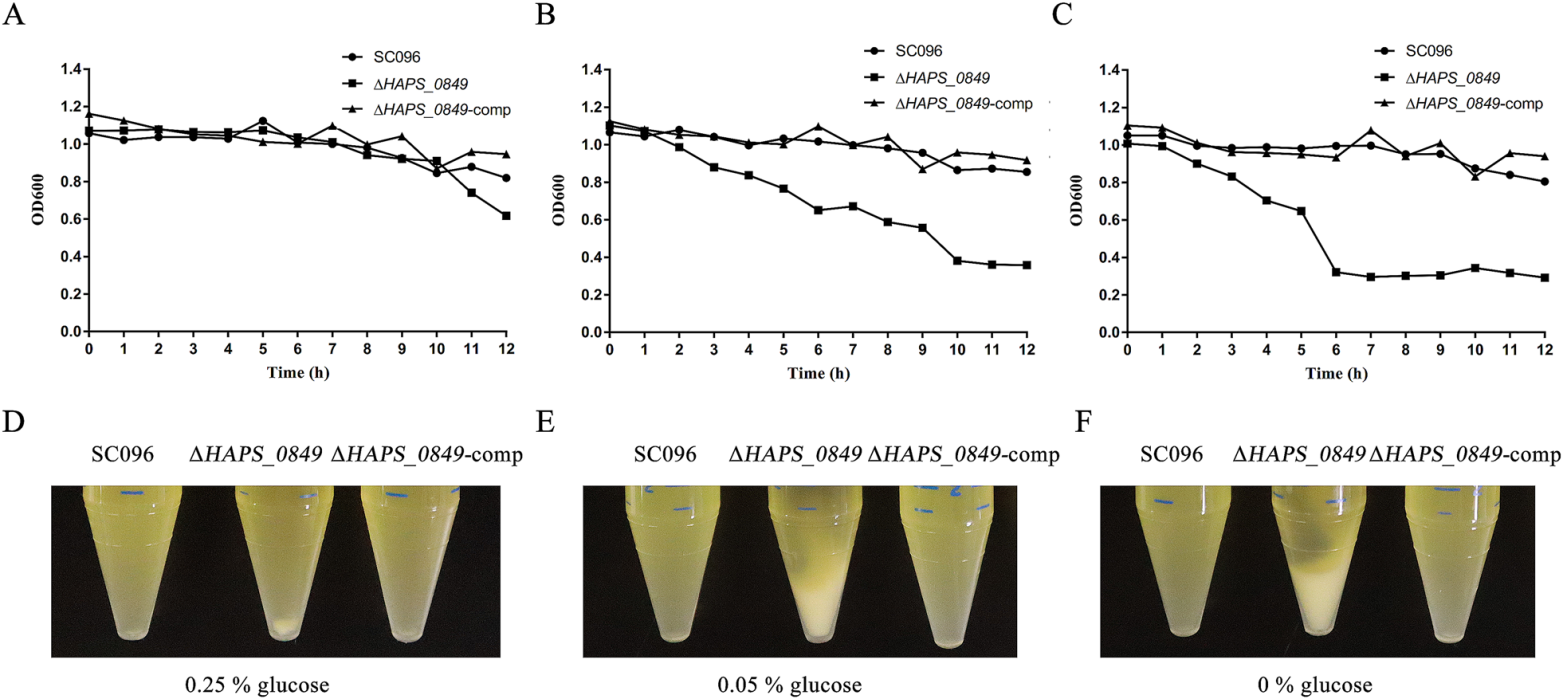
**Autoagglutination of*G. parasuis*SC096, Δ*HAPS_0849*and the*HAPS_0849*complementation strain. A** Autoagglutination rates of the SC096, Δ*HAPS_0849* and Δ*HAPS_0849*-comp strains cultured in TSB with 0.25% glucose. **D** A photograph was taken at the end of the assay, as shown in panel **A**, and the results were from a representative experiment. Lane 1, SC096; lane 2, *HAPS_0849* mutant; lane 3, complemented strain. **B**, **E** Assays were performed under the same conditions corresponding to panels **A** and **D** except that strains were cultivated in TSB broth with 0.05% glucose. **C**, **F** Tests were carried out in the same conditions corresponding to panels **A** and **D** except that strains were cultured without glucose. With the decrease in glucose, the rates of autoagglutination increased gradually.

The serum-sensitive phenotype and LOS truncation of Δ*HAPS_0849* indicate that *HAPS_0849* may be a virulence gene in *G. parasuis*. To further investigate the role of this gene, autoagglutination and biofilm formation assays were carried out as previously described. The aggregation results showed no obvious difference between SC096 and Δ*HAPS_0849* cultured in TSB with 0.25% glucose in the first 10 h; however, slight precipitation was observed after incubation was prolonged for 2 h (Figure 5A, D). The decrease in the optical density of Δ*HAPS_0849* dramatically accelerated when the strain was precultured in the same medium with a lower concentration (0.05%) of glucose (Figure 5B). Meanwhile, no apparent decrease in absorbance was observed in the wild-type and complemented strains. Furthermore, the aggregation of Δ*HAPS_0849* cultured in a lower glucose concentration was more obvious than that cultured in a higher glucose concentration, as shown in Figure 5E. The absorbance of Δ*HAPS_0849* at 600 nm drastically declined and reached its lowest value in 6 h when the strain was cultivated in TSB broth without glucose compared to the absorbance of SC096 (Figure 5C). The accumulation amount of Δ*HAPS_0849* cells in this condition was similar to that in 0.05% glucose after 12 h of incubation, while the phenotype of the complement strain was consistent with that of the parent strain (Figure 5F). The results indicated that the *HAPS_0849* mutant was inclined to autoagglutination and that the sedimentation rate was influenced by the concentration of glucose.

**Figure 6:**
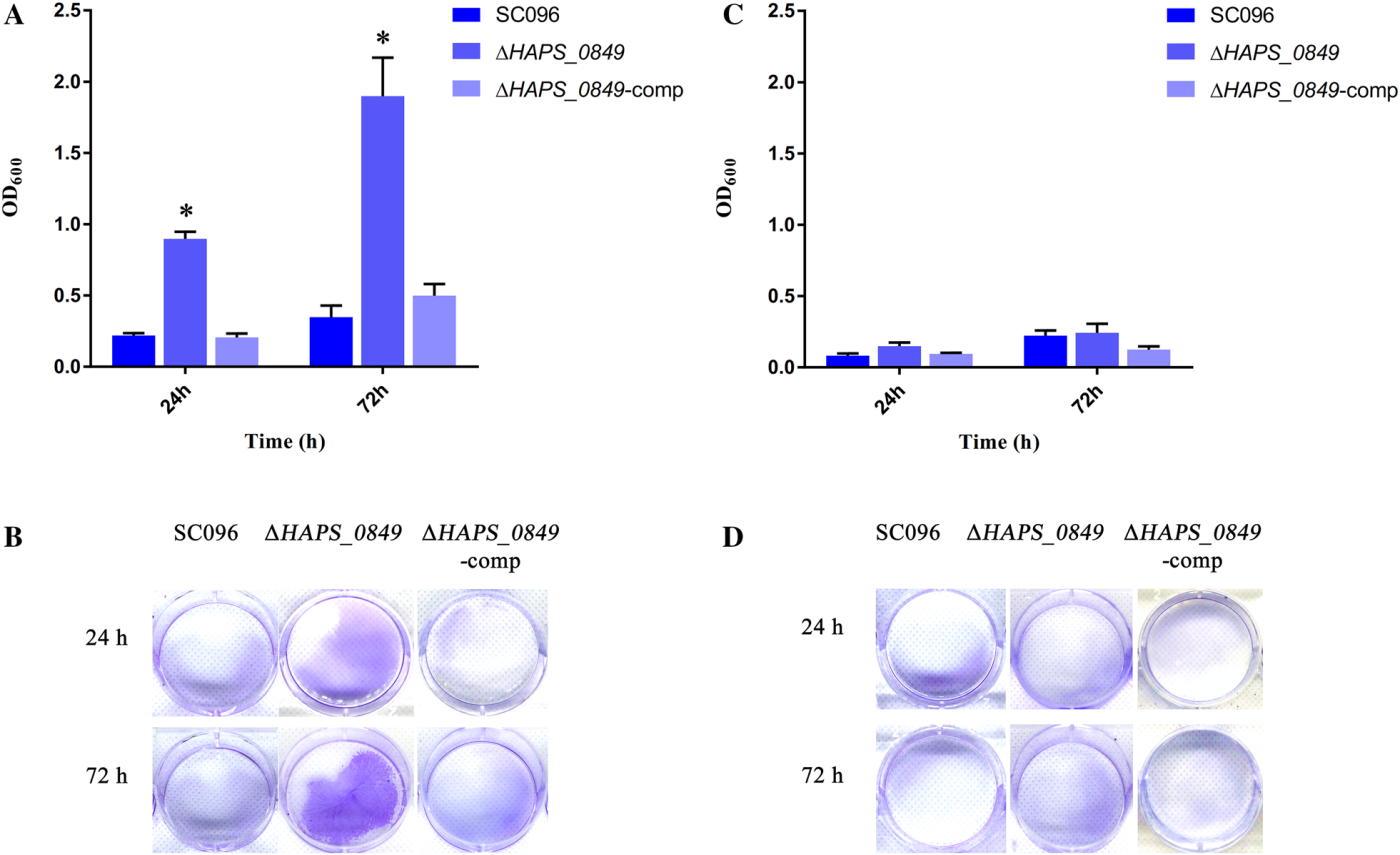
**Biofilm formation by SC096 and the*HAPS_0849*mutant and complementation strains. A** Quantification of biofilm production from 24-h or 72-h cultures in TSB broth without glucose. This graph indicated that disruption of *HAPS_0849* significantly increased the ability of the strain to form biofilms (*P < 0.05), while the biofilm formation phenotype of the complemented strain was restored to that of the wild-type strain SC096. **B** A photograph was taken at the end of the assay after cultivation in TSB with 0.25% glucose for 24 h or 72 h. **C**, **D** Experiments were performed under the same conditions except that strains were cultured with 0.25% glucose. These results showed that no obvious variance in the tested strains was observed when they were cultivated with adequate glucose. Error bars represent the standard deviations of three independent experiments.

The biofilm formation abilities of the wild-type and Δ*HAPS_0849* strains were assessed, and the results are depicted in Figure 6. After 24 h or 72 h, a significant gradual increase (*P* < 0.05) in biofilm biomass of Δ*HAPS_0849* was observed compared to the biofilm formed by SC096 or the complemented strain in the absence of glucose (Figure 6A, B). The absorbance of Δ*HAPS_0849* at 600 nm was close to 1.0 after 24 h and 2.0 after 72 h, while the absorbance values of the wild-type and complemented strains were less than 0.5 at both time points. The absorbance values of the stained biofilms of all strains did not exceed 0.5 when the strains were incubated in the presence of 0.25% glucose at different time intervals (Figure 6C, D). Hence, this evidence further supported the influence of glucose on the Δ*HAPS_0849* strain, and disruption of the *HAPS_0849* gene may increase biofilm formation in the absence of glucose.

### *HAPS_0849* may be an upper-pathway enzyme in uridine diphosphate galactose synthesis

**Figure 7:**
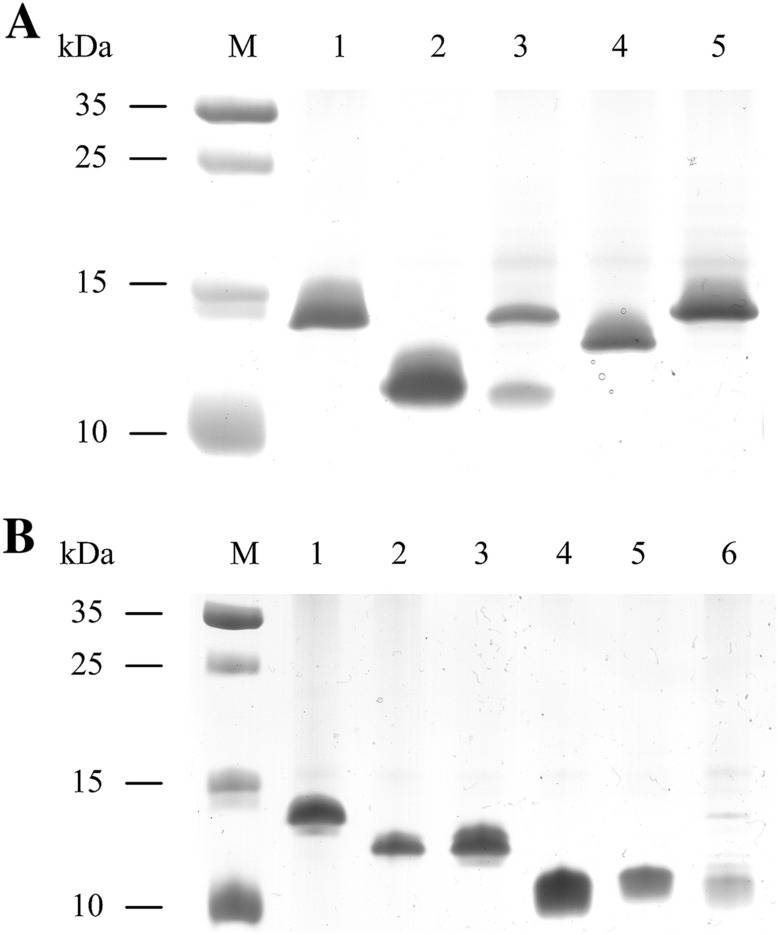
**Analysis of LOS profiles from mutants susceptible to serum. A** SDS-PAGE analysis of LOSs extracted from *G. parasuis* SC096, Δ*galU*, Δ*galE* and the corresponding complemented strains. Lane M, protein molecular marker; lane 1, SC096; lanes 2 and 3, Δ*galU* and its complemented strain Δ*galU*-c; lanes 4 and 5, Δ*galE* and its complemented strain. **B** Comparison of LOS profiles of serum-sensitive mutants. Lane M, protein molecular marker; lane 1, SC096; lane 2, Δ*galE*; lane 3, *lex*-*1*; lane 4, *galU*; lane 5, *lgtB*; lane 6, *HAPS_0849*.

We presented evidence that HAPS_0849 is a phosphoglucomutase that is responsible for producing G1P from G6P. Moreover, autoagglutination and biofilm phenotypes of Δ*HAPS_0849* indicated that *HAPS_0849* may correlate with surface polysaccharides biosynthesis. Thus, given our previous findings, we investigated whether *HAPS_0849* was an upper-pathway gene in the *galUE* pathway, as mentioned in “Introduction” section and Figure 1. Several mutants involved in polysaccharide synthesis were selected for LOS pattern comparison to explain the functional relationship between these genes. Two *galUE* pathway mutants of *G. parasuis* strain SC096, namely, ZY001 (*galU*) and ZY005 (*galE*), defective in serum resistance have previously been reported [17]. Analysis of the ZY001 and ZY005 LOSs by silver-stained PAGE revealed that loss of *galU* or *galE* alone in SC096 resulted in the truncation of the LOS core (Figure 7A). The LOS core from the *galU* mutant strain (lane 2) was smaller than that isolated from the *galE* mutant strain (lane 4). In general, the patterns of LOSs from the complemented strains were the same as those of the LOSs isolated from the parent strain SC096 (lanes 1, 3 and 5), except that the truncation of the LOS core was not completely eliminated in the Δ*galU*-complemented strain ZY002 (Δ*galU*-c) (lane 3), but the serum resistance phenotype was restored [17]. These findings suggested that *galU* and *galE* are required for LOS completeness, and disruption of *galU* or *galE* resulted in different LOS patterns.

Our previous data demonstrated that mutants of two additional glycosyltransferases, *lgtB* or *lex*-*1*, exhibited alterations in the LOS core [18]. To investigate the LOS patterns of these *G. parasuis* mutants, LOSs from the *galU*, *galE*, *lgtB*, *lex*-*1*, and *HAPS_0849* mutants and wild-type strain SC096 were extracted and visualized by SDS-PAGE. As shown in Figure 7B, the mutant lacking *galE* exhibited a LOS molecule (lane 2) with a migration band identical to that of the major glycoform produced by the *lex*-*1* mutant (lane 3). The LOSs from these two mutants migrated with an increased mobility compared to the LOS from the wild-type strain (lane 1) based on SDS-PAGE. This result suggests that *lex*-*1*, encoding a glycosyltransferase, may utilize UDP-galactose for LOS synthesis because *galE* is responsible for the production of this substrate. The *galU* mutant (lane 4) produced a highly truncated LOS molecule that migrated identically to the LOS produced by Δ*lgtB* (lane 5) or the lower band of Δ*HAPS_0849* (lane 6) and migrated more rapidly than the LOSs produced by Δ*galE*, Δ*lex*-*1* and the wild-type strain. It should be noted that all the tested mutants exhibited complete LOS truncation, except Δ*HAPS_0849*, and a small amount of wild-type LOS was produced by this strain.

Only two structures of LOSs from *G. parasuis* serovar 5 and serovar 15 have been previously reported, and they share the same structure [32] (Additional file 7). The completely deacylated LOS contains heptoses which are supposed to be transferred by *opsX*, *rfaF*, and *waaQ* [13] and multiple glucose and galactose provided by GalU for UDP-glucose and GalE for UDP-galactose, respectively. *galU* is also required for UDP-galactose synthesis because UDP-galactose is generated from UDP-glucose by GalE. This is consistent with the fact that the Δ*galU* strain produced a smaller LOS molecule than the *galE* mutant. We infer that GalU and HAPS_0849 may be contiguous in the UDP-galactose synthesis pathway, and then LgtB or Lex-1 transfer these glycosyl substrates to the LOS core. This finding may explain why there are mainly two types of LOS patterns, defects in both glucose and galactose or only a defect in galactose, in these mutants. Our previous studies showed that most of glycosyltransferases (GTs) genes such as *opsX*, *rfaF*, *waaQ*, *lgtB* and *lex*-*1* are involved in LOS synthesis [13, 18]. In order to confirm if all the GTs were participated in LOS formation, two GT genes *wcaJ* and *wclP* in *G. parasuis* were also chosen for LOS inspection. These two GT genes were predicted to lie in CPS synthesis locus, and their GenBank Accession numbers are KC795356 and KC795359 respectively. However, LOS from either mutant was intact as wild-type strain although these mutants were serum sensitive (Additional file 8).

Our data provide evidence that HAPS_0849 in *G. parasuis* may be an upper-pathway enzyme involved in the generation of glucose-1-phosphate; however, HAPS_0849 is not the only source of G1P based on the incomplete truncation of LOS in the *HAPS_0849* mutant strain.

## Discussion

PGM is responsible for the conversion of G6P to G1P and functions upstream of the *galUE* pathway. This protein is important for the virulence of a number of bacterial pathogens, including *Neisseria meningitidis* [33], *Brucella abortus* [34], *Bordetella bronchiseptica* [35] and *Salmonella enterica* serovar Typhimurium [36] and is a critical enzyme connecting glycolysis with the synthesis of polysaccharides, such as CPS, LPS and EPS (exopolysaccharides). Based on our previous findings that *galU* and *galE* contribute to serum resistance and biofilm formation in *G. parasuis* [17], we hypothesize that the *HAPS_0849* mutant may have similar phenotypes as the *galU* mutant based on the known pathway. However, the observation that the LOS pattern of Δ*HAPS_0849* was not completely consistent with that of Δ*galU* and shared a partially identical band with the LOS of the parent strain was unexpected. Unlike the phenotypes observed for Δ*galU*, glucose clearly affected autoagglutination and biofilm formation in the Δ*HAPS_0849* strain. This finding pointed out the main difference between Δ*galU* and the *HAPS_0849* mutant. The UDP-glucose generated by GalU appeared to be the sole source for LOS synthesis because the LOS in Δ*galU* was completely truncated. However, the substrate G1P converted by HAPS_0849 was not the only substrate according to our data provided here.

We highlight the importance of G1P because it is a central node involved in multiple carbon source pathways, including glycolysis, a central system of glucose metabolism. Because there are many phenotypes related to glucose, we suspect that there is another pathway to bypass the deficiency of G1P synthesis in the *HAPS_0849* mutant. G1P can be derived from at least four routes based on Kyoto Encyclopedia of Genes and Genomes (KEGG) database analysis. As shown in Additional file 9, (1) G1P can be derived from endogenous glycogen that can be degraded by glycogen phosphorylase (GlgP); however, it should be noted that endogenous glycogen is derived from G1P, and glycogen synthesis can occur only when G1P is present. (2) G1P can be derived from the metabolism of galactose, catalysed by galactokinase (GalK) and galactose-1-phosphate uridylyl transferase (GalT); however, no phenotypic variation was observed in mutants lacking these enzymes (data not shown). (3) G1P can be derived from exogenous glycogen, such as dextrin, which can also be converted by GlgP. The metabolic fingerprint of SC096 shows that dextrin is an ideal carbon source for *G. parasuis* (unpublished observation), and dextrin has been proposed as a potential source for G1P supplementation. Finally, (4) G1P can be derived from G6P produced by HAPS_0849, which should be the principal source for G1P generation according to the data presented above.

Several studies have demonstrated that CPS and LOS are important virulence factors and confer serum resistance in *G. parasuis* [5, 12, 13, 37]. However, the correlation between these polysaccharides and carbon source metabolism still awaits experimental investigation. Our previous work reported that two glycosyltransferases annotated as *lgtB* and *lex*-*1* are involved in the synthesis of LOS, and the absence of one of these genes can lead to a serum-sensitive phenotype [18]. Analysis of the LOSs of the mutants in this study showed that the LOS migration patterns of *galE* and *lex*-*1* were consistent, while the LOSs produced by *lgtB*, *HAPS_0849* and *galU* showed another glycoform. These results suggest that UDP-glucose may serve as a substrate for *lgtB*, while *lex*-*1* may use UDP-galactose as a donor for LOS synthesis, as the products of GalE and GalU are UDP-galactose and UDP-glucose, respectively. To date, only one study has performed structural analysis of the LOSs and CPSs of *G. parasuis*, and the results showed that multiple glucose and galactose residues do indeed exist in LOS molecules [32]. This finding may explain why the absence of *galU* or *galE* resulted in LOS truncation. In addition, these UDP sugars may also be involved in the synthesis of capsules because disruption of CPS genes reduced the resistance of *G. parasuis* to serum, and these genes are clearly not related to LOS expression. There are at least two types of polysaccharides that protect this bacterium from the effects of serum, and G1P could be a critical junction between glucose and UDP-sugars for these polysaccharides synthesis in *G. parasuis*.

In summary, we have identified *HAPS_0849* as a phosphoglucomutase gene that is correlated with the biosynthesis of LOS in *G. parasuis* SC096. The *HAPS_0849* mutant is not resistant to serum, and the complemented strain displayed a restored phenotype corresponding to that of the parent strain. Autoagglutination and biofilm formation were significantly increased in the Δ*HAPS_0849* strain. Moreover, the LOS profile of the *HAPS_0849* mutant was partially consistent with that of Δ*galU*, and *HAPS_0849* may act upstream of *galU* in the generation of G1P. Altogether, our observations highlight the fact that PGM is a key node for LOS synthesis in *G. parasuis*.

## Supplementary information

**Additional file 1. Bacterial strains used in this study.**

**Additional file 2. Plasmids used in this study.**

**Additional file 3. Sequences of the PCR primers used in this study.**

**Additional file 4. Construction of in-frame deletion and complementation strains of the*HAPS_0849*gene in*G. parasuis***. (A and B) Schematic diagram of the *HAPS_0849* mutant or complementation strain constructed in this study. Abbreviations: Kan^r^, kanamycin resistance gene; Gm^r^, gentamicin resistance gene; Up, upstream sequence of *HAPS_0849*; Dn, downstream sequence of *HAPS_0849*. (C) Locus structure of the *HAPS_0849* mutant or complementation strain. Detection primers are shown as solid black arrows. (D) PCR analysis confirming the constructs. Lane M, DNA molecular marker; lanes 1–3, SC096, Δ*HAPS_0849* and complementation strain with primers P21 and P22, as shown in this figure part C; lanes 4-6, the same strains with primers P23 and P24; lanes 7 and 8, Δ*HAPS_0849* and its complementation strain with primers P9 and P10.

**Additional file 5. Growth of SC096 and Δ*HAPS_0849***. Wild-type strain and Δ*HAPS_0849* were cultured in TSB broth containing 5% inactivated bovine serum and 0.005% NAD and supplemented with (A) or without (B) 0.25% glucose. Error bars represent the standard deviations of three independent experiments.

**Additional file 6. Construction of the*E. coli pgm*expression strain in the*HAPS_0849*mutant.** (A) Schematic diagram of the *HAPS_0849* mutant or Ecpgm expression strain constructed in this study. (B) Locus structure of the *HAPS_0849* mutant or Ecpgm expression strain. Detection primers are shown as solid black arrows. (C) PCR analysis confirming the constructs. Lane M, DNA molecular marker; lane 1, Δ*HAPS_0849*; and lane 2, Δ*HAPS_0849*-*ompP5*::*Ecpgm*.

**Additional file 7. LOS Structure of*G. parasuis***. Deacylated LOS of *G. parasuis* strains ER-6P and Nagasaki [32]. GalN, galactosamine; Gal, galactose; GlcN, Glucosamine; Glc, glucose; Kdo, 3-deoxy-D-manno-octulosonic acid; Hep, heptose. Dotted lines indicate potential attached site of different monosaccharides for glycosyltransferase LgtB (red) and Lex-1 (blue).

**Additional file 8. Construction and phenotype analysis of Δ*wclP*, Δ*wcaJ*and the corresponding complementation strains of*G. parasuis***. (A) PCR analysis verifying the Δ*wclP* constructs. Lane M, DNA molecular marker; lanes 1–3, SC096, Δ*wclP* and the complementation strain with primers P25 and P26, as shown in this figure part C; lanes 4–6, the same strains with primers P27 and P28; lanes 7 and 8, Δ*wclP* and its complementation strain with primers P9 and P10. (B) PCR analysis confirming the Δ*wcaJ* constructs. Lane M, DNA molecular marker; lanes 1–3, SC096, Δ*wcaJ* and the complementation strain with primers P29 and P30, as shown in this figure part C; lanes 4–6, the same strains with primers P31 and P32; lanes 7 and 8, Δ*wcaJ* and its complementation strain with primers P9 and P10. (C) Locus structure of *wclP*, *wcaJ* and the corresponding complementation strains. Detection primers are shown as black solid arrows. (D) Silver-stained SDS-PAGE gel of LOSs isolated from SC096 (lane 1), Δ*wclP* (lane 2) and Δ*wcaJ* (lane 3). (E) Survival of the *wclP* or *wcaJ* mutant treated with porcine serum. The Δ*wclP* or Δ*wcaJ* single-mutant strain exhibited significantly increased sensitivity to serum killing compared to the wild-type SC096 strain (*P* < 0.01). The complemented strain restored the serum resistance phenotype. Error bars represent the standard deviations from three independent experiments.

**Additional file 9. Proposed model of G1P formation and contributing to*G. parasuis*polysaccharide biosynthesis.** Glucose 1-phosphate can be generated from glucose, galactose and endogenous (glycogen) or exogenous (dextrin) glycogen, followed by conversion to UDP-glucose and UDP-galactose via GalU and GalE respectively. Both UDP-sugars then may participate in LOS or CPS formation.

## Data Availability

This work was supported by the National Natural Science Foundation of China (Grant no. 31802203 and no. 31772766) and by the Science and Technology Planning Project of Guangdong Province of China (Grant no. 2017B020233003).

## Abbreviations

*G. parasuis*: *Glaesserella parasuis*
*E. coli*: *Escherichia coli*
PGM: phosphoglucomutase
PMM: phosphomannomutase
TSA: trypticase soy agar
TSB: trypticase soy broth
NAD: nicotinamide adenine dinucleotide
G1P: glucose 1-phosphate
G6P: glucose 6-phosphate
UDP: uridine diphosphate
Gm: gentamicin
Km: kanamycin
NBS: newborn calf serum
MALDI-TOF–TOF: matrix-assisted laser desorption/ionization time-of-flight mass spectrometry
SDS-PAGE: sodium dodecyl sulphate-polyacrylamide gel electrophoresis
CPS: capsular polysaccharides
LOS: lipooligosaccharide
