# Supplementary material for: The Glaesserella parasuis phosphoglucomutase is partially required for lipooligosaccharide synthesis
Source: Vet Res. 2020 Jul 31;51:97. doi: 10.1186/s13567-020-00822-9 (PMC7393335; doi:10.1186/s13567-020-00822-9)
Supplement: Supplementary file 1 — Additional file 1. Bacterial strains used in this study. [file 13567_2020_822_MOESM1_ESM.docx]

**Additional file 1.** Bacterial strains used in this study

| **Strain** | **Relevant characteristic(s)** | **Source** |
| --- | --- | --- |
| ***E. coli* Strains** | |  |
| DH5α | F^-^, φ80d/*lacZ*ΔM15, Δ(*lacZYA*-*argF*) U169 *recA*1 *endA*1 *hsdR*17 | Laboratory collection |
| MG1655 | Wild-type strain | Laboratory collection |
| BL21 (DE3) | F^-^, *ompT hsdS_B_* (r_B_^-^, m_B_^-^) *gal dcm* (DE3) | Laboratory collection |
| SF419 | Expression strain of *HAPS_0849*, containing plasmid pET28- *HAPS_0849* in BL21 (DE3) | This study |
| ***G. parasuis* strains** | | |
| *G. parasuis* SC096 | Serovar 4 clinical isolate | [1] |
| QZ001 (Δ*lgtB*) | SC096 Δ*lgtB*::Kan^R^ | [2] |
| QZ002 (Δ*lex-1*) | SC096 Δ*lex-1*::Kan^R^ | [2] |
| ZY001 (Δ*galU*) | *galU*::ErmR derivative of SC096, Erm^R^ | [3] |
| ZY004 (Δ*galE*) | *galE*::ErmR derivative of SC096, Erm^R^ | [3] |
| ZY002 (Δ*galU*-c) | SC096 complemented Δ*galU* strain, Erm^R^ Kan^R^ | [3] |
| ZY005 (Δ*galE*-c) | SC096 complemented Δ*galE* strain, Erm^R^ Kan^R^ | [3] |
| SF412 (Δ*HAPS_0849*) | SC096 Δ*HAPS_0849*::Kan^R^ | This study |
| SF413 (Δ*wclP*) | SC096 Δ*wclP*::Kan^R^ | This study |
| SF414 (Δ*wcaJ*) | SC096 Δ*wcaJ*::Kan^R^ | This study |
| SF415 (Δ*HAPS_0849*-c) | SC096 complemented Δ*HAPS_0849* strain, Gm^R^ Kan^R^ | This study |
| SF416 (Δ*wclP*-c) | SC096 complemented Δ*wclP* strain, Gm^R^ Kan^R^ | This study |
| SF417 (Δ*wcaJ*-c) | SC096 complemented Δ*wcaJ* strain, Gm^R^ Kan^R^ | This study |
| SF418 (Δ*HAPS_0849*-*Ecpgm*) | SC096 complemented Δ*HAPS_0849* strain with *Ecpgm*, Gm^R^ Kan^R^ | This study |

**References**

[1] Zhang B., Feng S., Xu C., Zhou S., He Y., Zhang L., Zhang J., Guo L., Liao M., Serum resistance in *Haemophilus parasuis* SC096 strain requires outer membrane protein P2 expression, FEMS Microbiol Lett. (2012) 326:109-115.

[2] Zhou Q., Feng S., Zhang J., Jia A., Yang K., Xing K., Liao M., Fan H., Two Glycosyltransferase Genes of *Haemophilus parasuis* SC096 Implicated in Lipooligosaccharide Biosynthesis, Serum Resistance, Adherence, and Invasion, Front Cell Infect Microbiol. (2016) 6:100.

[3] Zou Y., Feng S., Xu C., Zhang B., Zhou S., Zhang L., He X., Li J., Yang Z., Liao M., The role of *galU* and *galE* of *Haemophilus parasuis* SC096 in serum resistance and biofilm formation, Vet Microbiol. (2013) 162:278-284.
