# Supplementary material for: The Glaesserella parasuis phosphoglucomutase is partially required for lipooligosaccharide synthesis
Source: Vet Res. 2020 Jul 31;51:97. doi: 10.1186/s13567-020-00822-9 (PMC7393335; doi:10.1186/s13567-020-00822-9)
Supplement: Supplementary file 2 — Additional file 2. Plasmids used in this study. [file 13567_2020_822_MOESM2_ESM.docx]

**Additional file 2.** Plasmids used in this study

| **Plasmid** | **Relevant characteristic(s)** | **Source** |
| --- | --- | --- |
| pMD19-T | Cloning vector, Amp^R^ | Takara Inc. |
| pK18mobsacB | Suicide and narrow-broad-host vector, Kan^R^ | [1] |
| pET28 (a) | Expression vector, Kan^R^ | Novagen |
| pSF116 | Gm resistance cassette-carrying complement vector, Gm^R^ | [2] |
| pSF251 | A disruption cassette containing KanR, the upstream and downstream sequences of *HAPS_0849* in pMD19-T, Amp^R^ Kan^R^ | This study |
| pSF252 | A disruption cassette containing KanR, the upstream and downstream sequences of *wclP* in pMD19-T, Amp^R^ Kan^R^ | This study |
| pSF253 | A disruption cassette containing KanR, the upstream and downstream sequences of *wcaJ* in pMD19-T, Amp^R^ Kan^R^ | This study |
| pSF254 | *HAPS_0849* gene cloned in pSF116 with SphI and SalI, Gm^R^ | This study |
| pSF255 | *wclP* gene cloned in pSF116 with SphI and SalI, Gm^R^ | This study |
| pSF256 | *wcaJ* gene cloned in pSF116 with SphI and SalI, Gm^R^ | This study |
| pSF257 | *E. coli* *pgm* gene cloned in pSF116 with SphI and SalI, Gm^R^ | This study |
| pSF258 | *HAPS_0849* gene cloned in pET28 (a) with NdeI and HindIII | This study |

**References**

[1] Schafer A., Tauch A., Jager W., Kalinowski J., Thierbach G., Puhler A., Small mobilizable multi-purpose cloning vectors derived from the *Escherichia coli* plasmids pK18 and pK19: selection of defined deletions in the chromosome of *Corynebacterium glutamicum*, Gene. (1994) 145:69-73.

[2] Feng S., Xu C., Yang K., Wang H., Fan H., Liao M., Either *fadD1* or *fadD2*, Which Encode acyl-CoA Synthetase, Is Essential for the Survival of *Haemophilus parasuis* SC096, Front Cell Infect Microbiol. (2017) 7:72.
