# Supplementary material for: The Glaesserella parasuis phosphoglucomutase is partially required for lipooligosaccharide synthesis
Source: Vet Res. 2020 Jul 31;51:97. doi: 10.1186/s13567-020-00822-9 (PMC7393335; doi:10.1186/s13567-020-00822-9)
Supplement: Supplementary file 3 — Additional file 3. Sequences of the PCR primers used in this study. [file 13567_2020_822_MOESM3_ESM.docx]

**Additional file 3.** Sequences of the PCR primers used in this study

| **Primers** | **Primer sequences (5′-3′)** |
| --- | --- |
| P1 (*HAPS_0849*-Uf for mut) | ACCgCTTgTgTCTTgAAgCggCTAAAgTTg |
| P2 (*HAPS_0849*-Ur for mut) | ATgTCAATTCgggATCCgCgTAAgTTTTgATgCCTgCTgC |
| P3 (*HAPS_0849*-Df for mut) | gATCggCTTCgTCgACACgTAgCCgAAAATCAAgTTCTAC |
| P4 (*HAPS_0849*-Dr for mut) | TTCACTggCTCgATTATTAC |
| P5 (*wclP*-Uf for mut) | ACCgCTTgTgTCTTgCCTATATCTATTCTAGAACG |
| P6 (*wclP*-Ur for mut) | ATgTCAATTCgggATCCgCgATTCAGAAAGGGAATATCTC |
| P7 (*wclP*-Df for mut) | GATCGGCTTCgtcgacacgtTCTCATCCTGAAGATTTATC |
| P8 (*wclP*-Dr for mut) | GGTTTCCAATCACATTAGAT |
| P9 (pSF116-F) | ATCgTTTgCTTAgCACCAgA |
| P10 (pSF116-R) | ACAggATTACgCTTCTgCTT |
| P11 (Km-F) | CGCGGATCCCGAATTGACATTTTTATGGACAGCAAGCGAA |
| P12 (Km-R) | ACGTGTCGACGAAGCCGATCTCAGAAGAACTCGTCAAGAA |
| P13 (*HAPS_0849*-F for comp) | CGCGTCGACAACTggATCgTATTgCTCAA |
| P14 (*HAPS_0849*-R for comp) | CATGCATGCTgAAAAgTgTCggTAATAgA |
| P15 (*wclP*-F for comp) | CGCGTCGACTCTACTAATGTTGGAGGATG |
| P16 (*wclP*-R for comp) | CATGCATGCGTGGAATCATTAGAAGCATC |
| P17 (*wcaJ*-F for comp) | CGCGTCGACCAATATATTTTTTCAGATGATAGTAATG |
| P18 (*wcaJ*-R for comp) | CATGCATGCCTACCTTgCACCAAATCCAg |
| P19 (*Ecpgm*-F for comp) | CGCGTCGACAAGGACAAAGCAATggCAATCCACAATCgTgC |
| P20 (*Ecpgm*-R for comp) | CATGCATGCTTACgCgTTTTTCAgAACTTCg |
| P21 (*HAPS_0849*-testin-F) | TgCCTATgCAATCAAgTACT |
| P22 (*HAPS_0849*-testin-R) | gTgCATCTggTACTgCCACT |
| P23 (*HAPS_0849*-testout-F) | AACTggATCgTATTgCTCAA |
| P24 (*HAPS_0849*-testout-R)  P25 (*wclP*-testin-F) | TgAAAAgTgTCggTAATAgA |
|  | TgATgCTATTgATggAAgAg |
| P26 (*wclP*-testin-R) | TCTTTCCACAACAgCTCTAg |
| P27 (*wclP*-testout-F) | ACTAATgTTggAggATgTAC |
| P28 (*wclP*-testout-R) | CTAgATgATgATACACCTTC |
| P29 (*wcaJ*-testin-F) | AgCTgAAACTgCgTTAATgA |
| P30 (*wcaJ*-testin-R) | ATACTTTCTgTTCTggCCgT |
| P31 (*wcaJ*-testout-F) | TTATCCTggTAACCgTTCTg |
| P32 (*wcaJ*-testout-R) | AATAggAgATTgTgTCgTAg |
| P33 (*wcaJ*-Uf for mut) | ACCgCTTgTgTCTTgTAATCAAGTGAATGCTAACC |
| P34 (*wcaJ*-Ur for mut) | ATGTCAATTCGggatccgcgATGATAGGAAATATAACGTC |
| P35 (*wcaJ*-Df for mut) | GATCGGCTTCgtcgacacgtTTGGTTAGATATTCTCATTG |
| P36 (*wcaJ*-Dr for mut) | TTTAATATGTCGCCTACACC |
| P37 (*OmpA*-testout-F) | ttccgtgttgagtaccaatg |
| P38 (*OmpA*-testout-R) | agaataattggtaacaaacc |
| P39 (*HAPS_0849*-pET-F) | CCGCATATGGAAACTCTCTTTCAAGT |
| P40 (*HAPS_0849*-pET-R) | CCGAAGCTTCTAGCAATCTTGTTTACCA |
