# Supplementary figures and images for: The Glaesserella parasuis phosphoglucomutase is partially required for lipooligosaccharide synthesis
Source: Vet Res. 2020 Jul 31;51:97. doi: 10.1186/s13567-020-00822-9 (PMC7393335; doi:10.1186/s13567-020-00822-9)

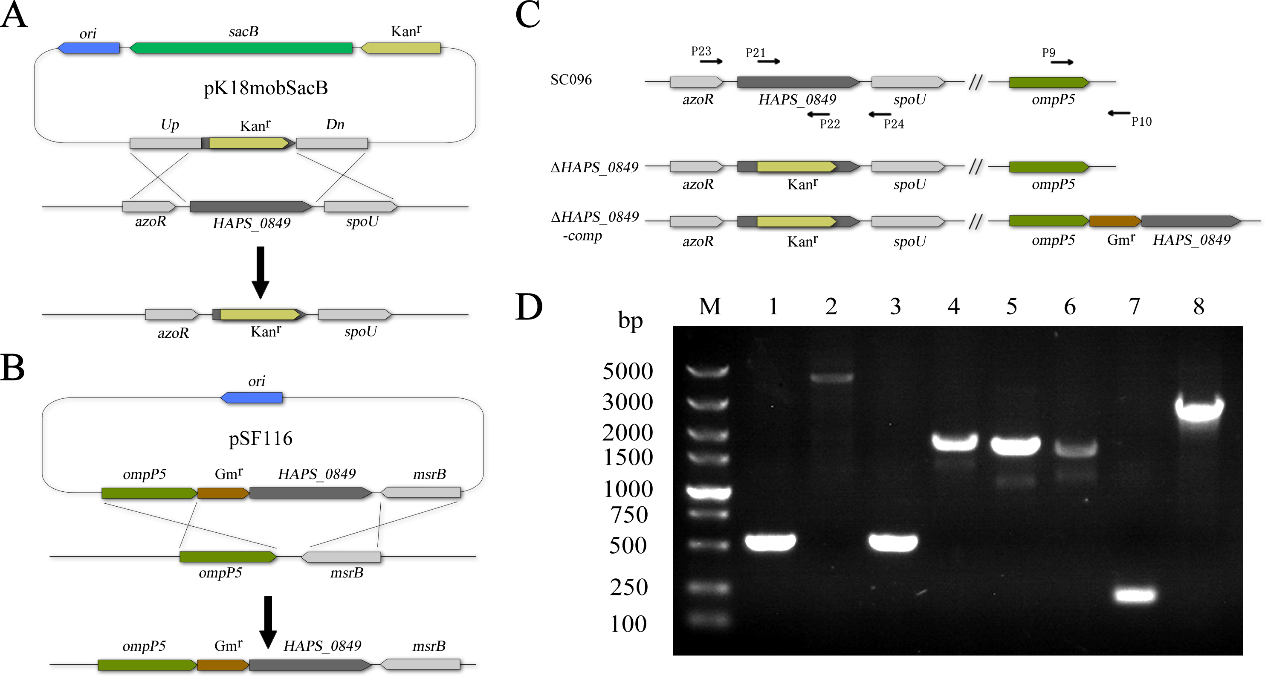

Supplement: Supplementary file 4 — Additional file 4. Construction of in-frame deletion and complementation strains of theHAPS_0849gene inG. parasuis. (A and B) Schematic diagram of the HAPS_0849 mutant or complementation strain constructed in this study. Abbreviations: Kanr, kanamycin resistance gene; Gmr, gentamicin resistance gene; Up, upstream sequence of HAPS_0849; Dn, downstream sequence of HAPS_0849. (C) Locus structure of the HAPS_0849 mutant or complementation strain. Detection primers are shown as solid black arrows. (D) PCR analysis confirming the constructs. Lane M, DNA molecular marker; lanes 1–3, SC096, ΔHAPS_0849 and complementation strain with primers P21 and P22, as shown in this figure part C; lanes 4-6, the same strains with primers P23 and P24; lanes 7 and 8, ΔHAPS_0849 and its complementation strain with primers P9 and P10. [file 13567_2020_822_MOESM4_ESM.docx]

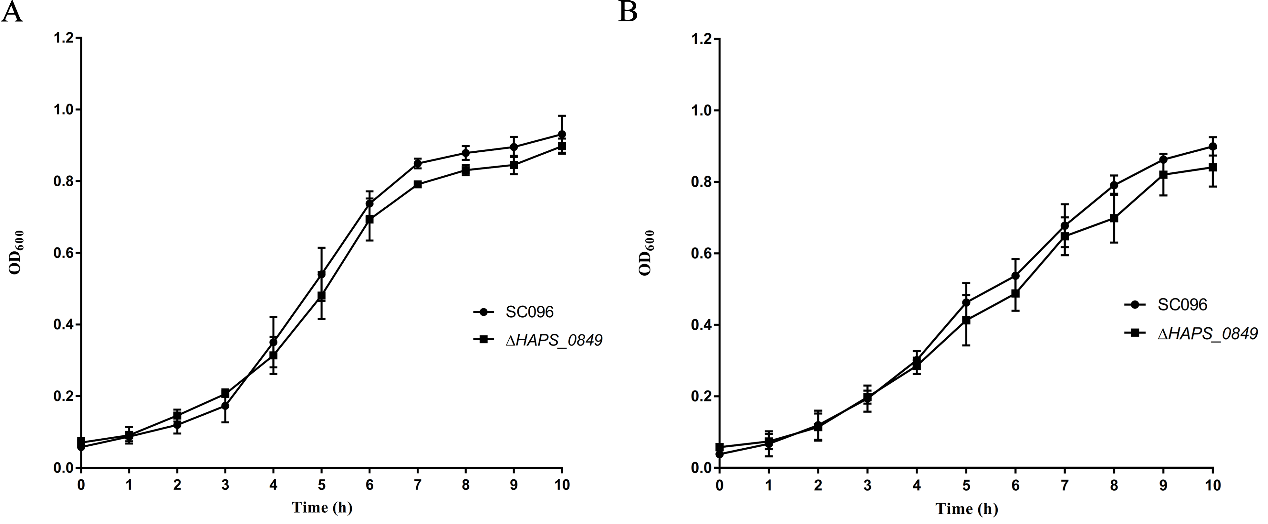

Supplement: Supplementary file 5 — Additional file 5. Growth of SC096 and ΔHAPS_0849. Wild-type strain and ΔHAPS_0849 were cultured in TSB broth containing 5% inactivated bovine serum and 0.005% NAD and supplemented with (A) or without (B) 0.25% glucose. Error bars represent the standard deviations of three independent experiments. [file 13567_2020_822_MOESM5_ESM.docx]

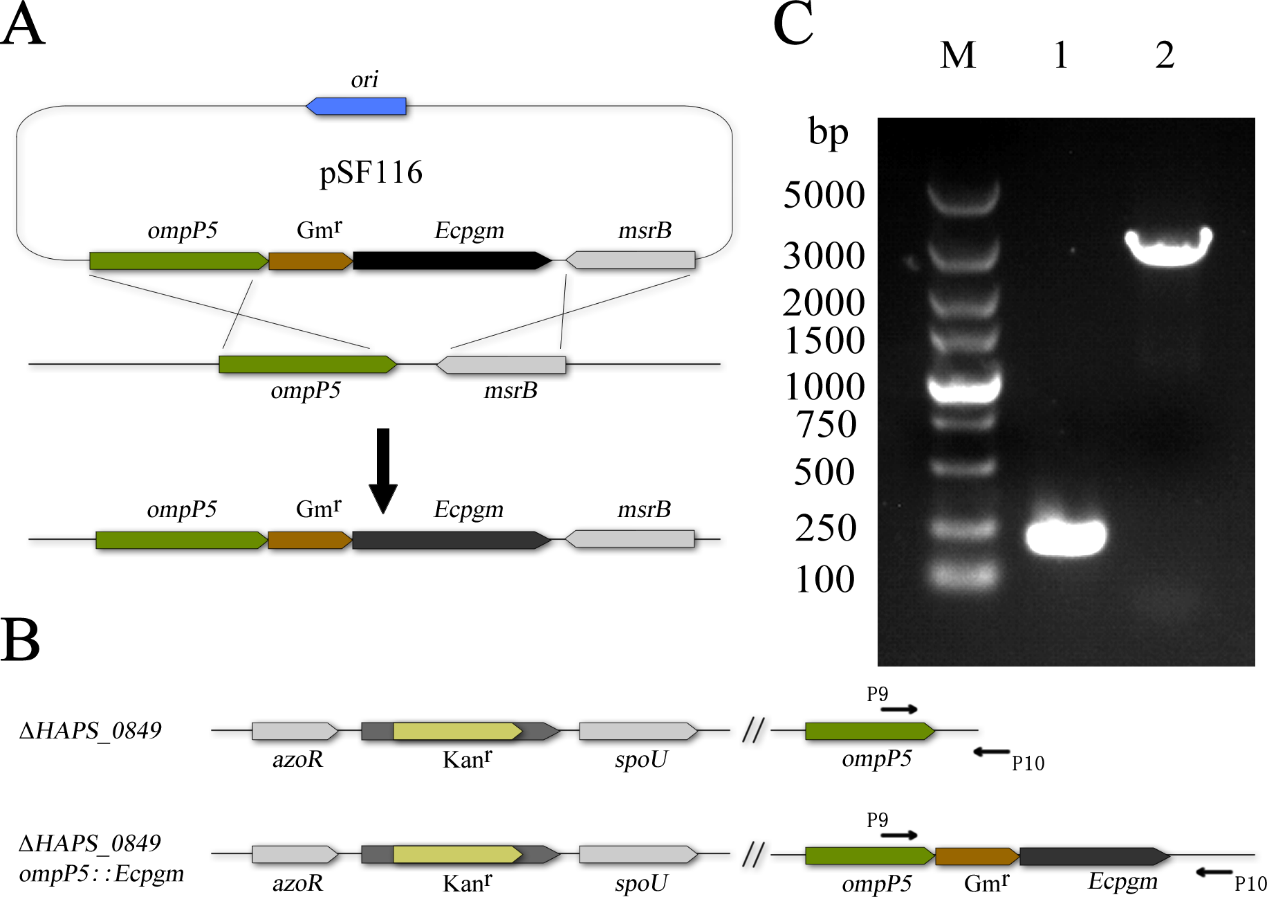

Supplement: Supplementary file 6 — Additional file 6. Construction of theE. coli pgmexpression strain in theHAPS_0849mutant. (A) Schematic diagram of the HAPS_0849 mutant or Ecpgm expression strain constructed in this study. (B) Locus structure of the HAPS_0849 mutant or Ecpgm expression strain. Detection primers are shown as solid black arrows. (C) PCR analysis confirming the constructs. Lane M, DNA molecular marker; lane 1, ΔHAPS_0849; and lane 2, ΔHAPS_0849-ompP5::Ecpgm. [file 13567_2020_822_MOESM6_ESM.docx]

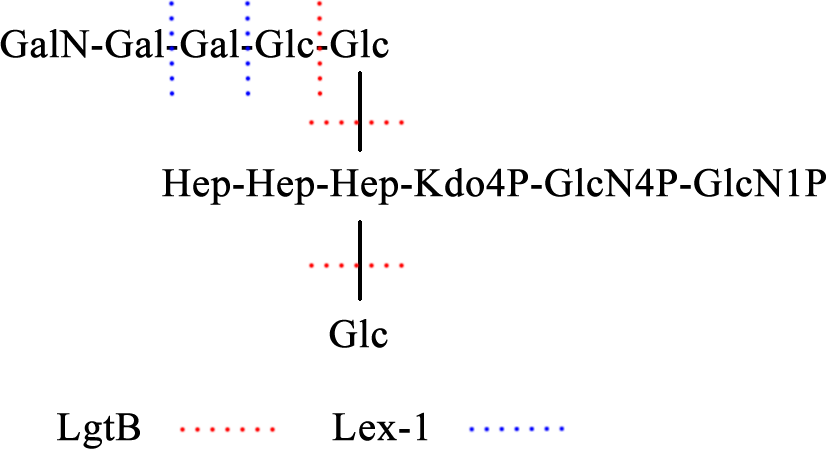

Supplement: Supplementary file 7 — Additional file 7. LOS Structure ofG. parasuis. Deacylated LOS of G. parasuis strains ER-6P and Nagasaki [32]. GalN, galactosamine; Gal, galactose; GlcN, Glucosamine; Glc, glucose; Kdo, 3-deoxy-D-manno-octulosonic acid; Hep, heptose. Dotted lines indicate potential attached site of different monosaccharides for glycosyltransferase LgtB (red) and Lex-1 (blue). [file 13567_2020_822_MOESM7_ESM.docx]

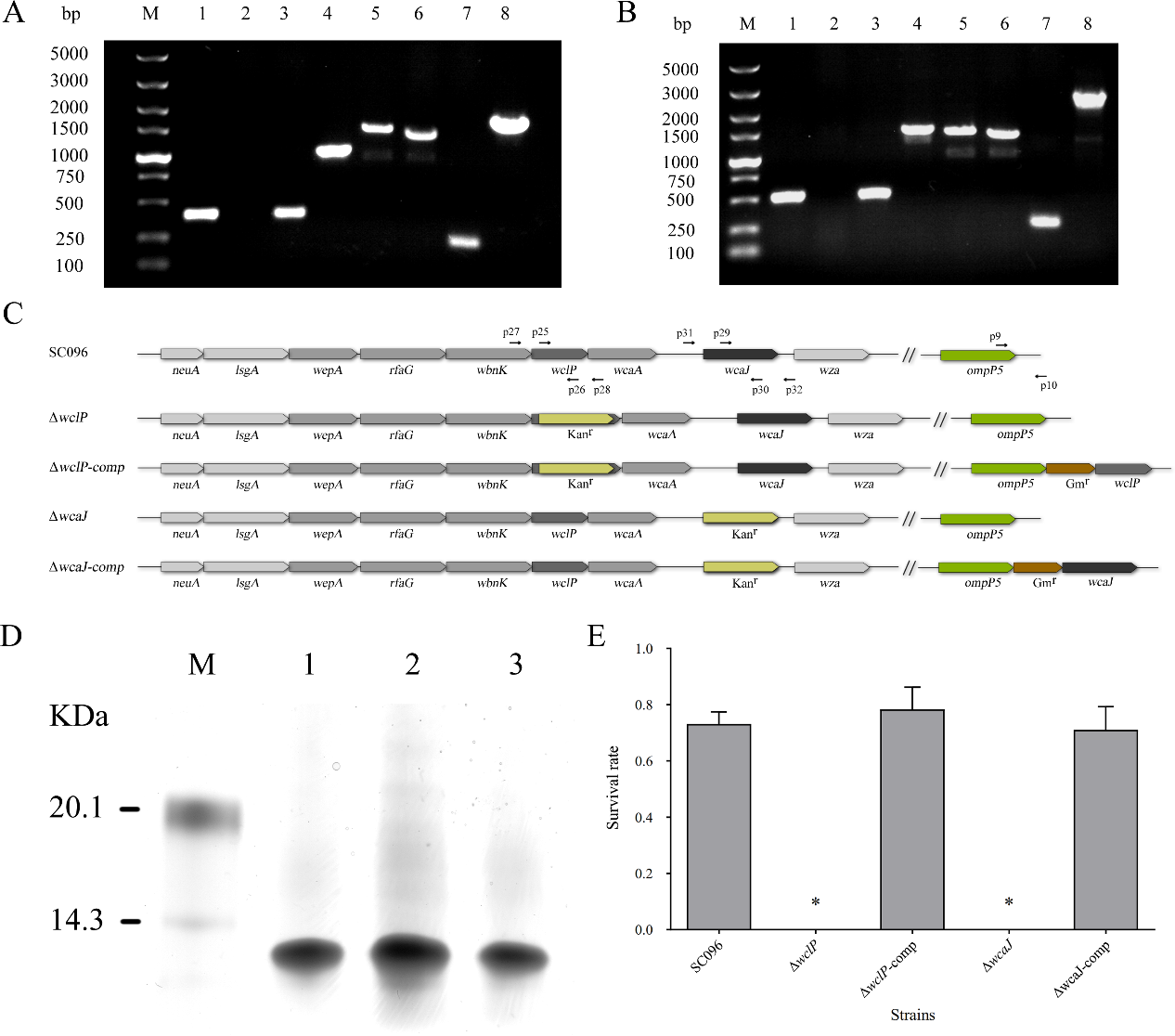

Supplement: Supplementary file 8 — Additional file 8. Construction and phenotype analysis of ΔwclP, ΔwcaJand the corresponding complementation strains ofG. parasuis. (A) PCR analysis verifying the ΔwclP constructs. Lane M, DNA molecular marker; lanes 1–3, SC096, ΔwclP and the complementation strain with primers P25 and P26, as shown in this figure part C; lanes 4–6, the same strains with primers P27 and P28; lanes 7 and 8, ΔwclP and its complementation strain with primers P9 and P10. (B) PCR analysis confirming the ΔwcaJ constructs. Lane M, DNA molecular marker; lanes 1–3, SC096, ΔwcaJ and the complementation strain with primers P29 and P30, as shown in this figure part C; lanes 4–6, the same strains with primers P31 and P32; lanes 7 and 8, ΔwcaJ and its complementation strain with primers P9 and P10. (C) Locus structure of wclP, wcaJ and the corresponding complementation strains. Detection primers are shown as black solid arrows. (D) Silver-stained SDS-PAGE gel of LOSs isolated from SC096 (lane 1), ΔwclP (lane 2) and ΔwcaJ (lane 3). (E) Survival of the wclP or wcaJ mutant treated with porcine serum. The ΔwclP or ΔwcaJ single-mutant strain exhibited significantly increased sensitivity to serum killing compared to the wild-type SC096 strain (P < 0.01). The complemented strain restored the serum resistance phenotype. Error bars represent the standard deviations from three independent experiments. [file 13567_2020_822_MOESM8_ESM.docx]

**
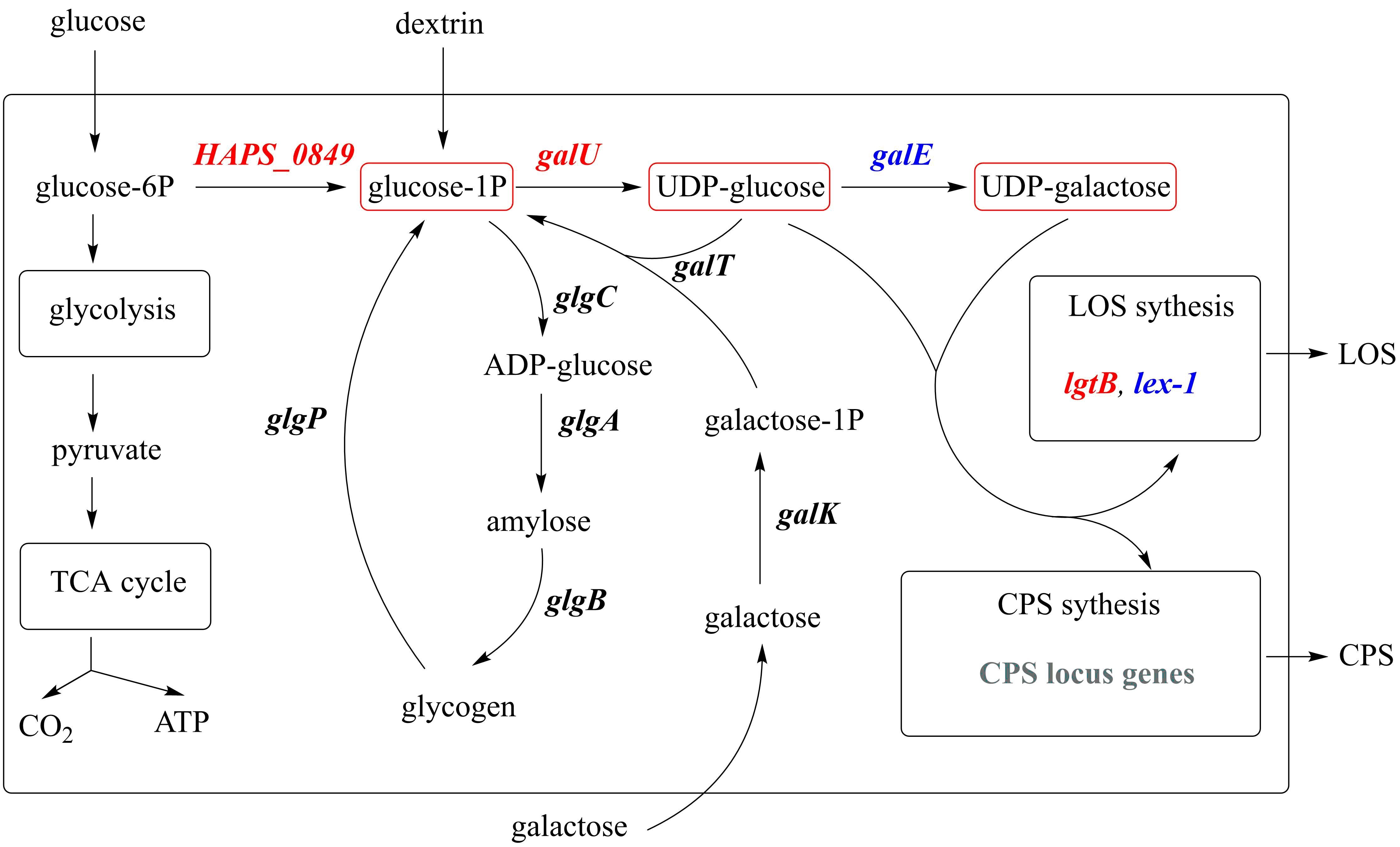
**

Supplement: Supplementary file 9 — Additional file 9. Proposed model of G1P formation and contributing toG. parasuispolysaccharide biosynthesis. Glucose 1-phosphate can be generated from glucose, galactose and endogenous (glycogen) or exogenous (dextrin) glycogen, followed by conversion to UDP-glucose and UDP-galactose via GalU and GalE respectively. Both UDP-sugars then may participate in LOS or CPS formation. [file 13567_2020_822_MOESM9_ESM.docx]
